# Supplementary material for: Structural and practical identifiability of contrast transport models for DCE-MRI
Source: PLoS Comput Biol. 2024 May 15;20(5):e1012106. doi: 10.1371/journal.pcbi.1012106 (PMC11132485; doi:10.1371/journal.pcbi.1012106)
Supplement: S1 Text — It contains Fig A. (PDF) [file pcbi.1012106.s001.pdf]

S1 Identifiability of PM, TK and eTK models

Here, we report the results of structural (S1.1 Text) and practical (S1.2 Text) identifiability of Patlak, Tofts–Kety, and extended Tofts–Kety models. These belong to the same family of nested compartment models to which LTK belongs and from which it has been derived. A complete description of their structure and related references have been provided in Section 2.2.

S1.1 Structural identifiability for PM, TK, and eTK models

Assuming the leakage compartment and the reverse flux from extracellular space to the plasma space is negligible (i.e., $\lambda \approx 0$ and $C_e(t)$ evolution given by Eq. (4) in the manuscript), from the LTK model we reduce to the Patlak model. Using the formalism of the differential algebra approach introduced in Section 2.4, the PM can be written as

$$\begin{cases} v_e \dot{x} = K^{trans} u \\ y = v_e x + v_p u \end{cases}$$

where y is the observable concentration of the contrast agent in the tissue $C_t(t)$ and $u(t)$ is the external input given by the concentration of contrast agent in the plasma compartment (VIF). The state variable for the CA concentration into the EES compartment is given by x. Differentiating the second equation and combining it with the first, we obtain the differential equation for $y(t)$ in the form

$$\dot{y} + a_1 u + a_2 \dot{u} = 0$$

where

$$a_1 = -K^{trans} \quad \text{and} \quad a_2 = -v_p \,. \tag{S.1}$$

Thus, both parameters of the PM are structurally identifiable, allowing us to conclude that PM model is structurally identifiable.

The TK model can be obtained from the LTK by assuming that both leakage and intravascular compartment contributions are negligible (i.e., $v_p, \lambda \approx 0$). Using the same differential algebra approach, TK can be written as

$$\begin{cases} v_e \dot{x} = K^{trans} \left(u - \dfrac{x}{v_e} \right) \\ y = v_e x \,. \end{cases}$$

This is analogous to the PM, as these two equations can be combined into the following equation for $y(t)$:

$$\dot{y} + a_1 y + a_2 u = 0$$

with

$$a_1 = \frac{K^{trans}}{v_e^2} \quad \text{and} \quad a_2 = -K^{trans} \,. \tag{S.2}$$

Thus, the TK model is structurally identifiable, as both K^{trans} and v_e are structurally identifiable.

Finally, the eTK model can be derived from LTK assuming that leakage compartment contribution is negligible (i.e., $\lambda \approx 0$). In the differential algebra formalism, eTK reads as

$$\begin{cases} v_e \dot{x} = K^{trans} \left(u - \dfrac{x}{v_e} \right) \\ y = v_e x + v_p u \end{cases}$$

whose equations can be recombined in the following expression for the evolution of the observable $y(t)$

$$\dot{y} + a_1 y + a_2 u + +a_3 \dot{u} = 0$$

with

$$\begin{cases} a_1 = K^{trans}/v_e^2 \\ a_2 = -K^{trans} \\ a_3 = -v_p \,. \end{cases} \tag{S.3}$$

Therefore eTK is also structurally identifiable, since all of its parameters are structurally identifiable.

S1.2 Practical identifiability for PM, TK, and eTK models

Practical identifiability is performed using the method described in Section 2.4.2. As done for LTK model, each of these models has been analyzed in the three cases of study (AA), (RA), and (RR), described in 3.2, and for the three different time-enhancement profiles observed in CA evolution. For the purpose of this study and in analogy with LTK analysis, here we report the results only for the Type I time-enhancement curve and for the parameter K^{trans}. Fig A collects the results concerning practical identifiability of this parameter for PM (A-**A**), TK (A-**B**), and eTK (A-**C**) models for Type I time-enhancement curve.

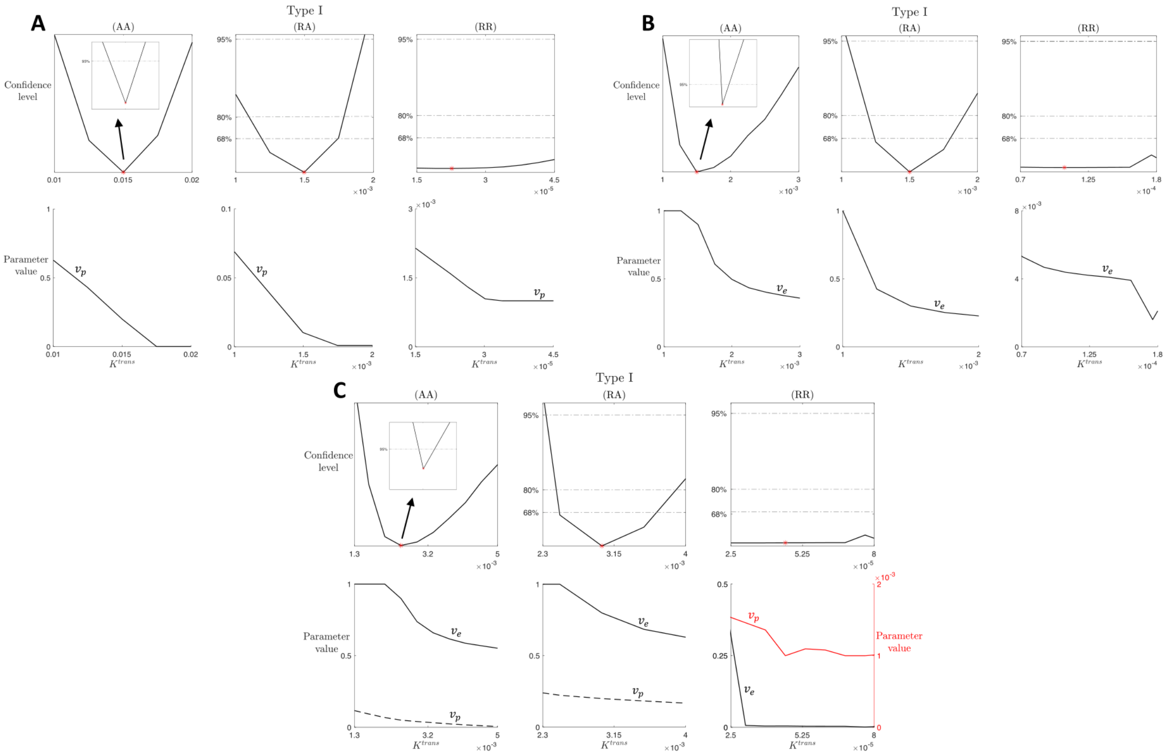

Figure A: K^{trans} **practical identifiability for PM (A), TK (B), and eTK (C) models and Type I enhancement curve.** Top row in **A**, **B**, and **C**: profile likelihood and confidence levels at 68%, 80%, and 95% for the parameter K^{trans} in the (AA), (RA), and (RR) case for the Type I enhancement curve. Inset in the first subplot shows a zoom of the region around the best-fitted value $\hat{K}^{trans}$ (red marker). Bottom row in **A**, **B**, and **C**: compensating profiles of the parameters v_p (in **A**), v_e (in **B**), and v_e and v_p (in **C**) with respect to variation of K^{trans} around its best-fitted value. Variation of $\pm 50\%$ around the optimal value of K^{trans} are considered. Two colors are for two different y-axis (when needed): black curves refer the left y-axis and red curves to the right y-axis. Different line styles are used to distinguish curves referring to the same y-axis. For each curve, the name of the corresponding parameter is indicated above the line in the same color.

The columns of each panel of Fig A refer to the three cases of study (AA), (RA), and (RR), the top rows show the profile likelihood, and the bottom rows the compensating profiles for the other parameters involved in the models. From the structural identifiability analysis of S1.1 Text, we know that K^{trans} is structurally identifiable in PM, TK, and eTK, or equivalently, it is possible to uniquely obtain its expression from the given model structures. The columns referring to the (AA) case in **A**, **B**, and **C**, show that when an artificial data set is used, the practical identifiability of K^{trans} is possible. In fact, the profile likelihoods show in all cases a well-defined parabola, with a unique minimum in the optimal value $\hat{K}^{trans}$ and the zoomed inset around this minimum shows a finite confidence region for the 95% confidence level. Analyzing the (RA) case (second columns in **A**, **B**, and **C**), instead, we notice that the confidence level for the identifiability of K^{trans} in the three models decreases and we get a finite confidence region only for the 80% level, while it is not possible to define the lower/upper bound of the confidence region for 95% confidence level. This is in line with the results concerning the influence of VIF noise on parameter identifiability shown in Fig 6. Finally, looking at the (RR) results (third columns in **A**, **B**, and **C**), as for LTK model, we obtain flat evolution for the profile likelihood, meaning that the parameter K^{trans} is practically non-identifiable. Compensating profiles (in the bottom rows of **A**, **B**, and **C**) show the response of the other model parameters to K^{trans} variability around its best value. In particular, looking at eTK model (in **C**) we observe that v_p has a rather small response to K^{trans} variation,

while v_e seems to show more evident changes in response to K^{trans}. This result is reasonable in relation to what is shown in results in Eq. (S.3): in fact, the expression for v_p is independent from the others.
