# Supplementary material for: Structural and practical identifiability of contrast transport models for DCE-MRI
Source: PLoS Comput Biol. 2024 May 15;20(5):e1012106. doi: 10.1371/journal.pcbi.1012106 (PMC11132485; doi:10.1371/journal.pcbi.1012106)
Supplement: S2 Text — It contains Figs A and B. (PDF) [file pcbi.1012106.s002.pdf]

S2 modified Leaky Toft-Kety (mLTK): a structurally non-identifiable model

Here, we illustrate an example of a structurally non-identifiable model for the analysis of DCE-MRI data, showing the results about both structural and practical identifiability of its parameters. It belongs to the family of nested compartmental models on which the proposed analysis is focused and it represents a modified version of the LTK model described in Section 2.2. An illustration of this mLTK model is shown in Fig A.

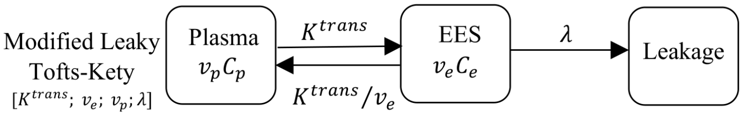

Figure A: **Scheme for mLTK model (S.4).** Schematic illustration of the modified Leaky Tofts–Kety model: the contrast agent concentration $C_t(t)$ is evaluated using the functions $C_p(t)$, the CA concentration in the plasma compartment, which is assumed to be given by the arterial input function, and $C_e(t)$, for the CA concentration in the EES space. The rate of forward and backward volume transfer and the fractional EES and plasma volumes are the quantifies K^{trans}, v_e, v_p, and λ. The model parameters are listed in brackets.

As with the LTK model, the mLTK includes a further compartment, the leakage compartment, which accounts for the loss of CA concentration that, coming out from the EES compartment does not flow back into the plasma compartment. Separately from the LTK, we do not consider a direct flow between PS and leakage compartment, but only a unidirectional flow from EES to the leakage compartment. Thus, the concentration of CA into the EES compartment varies according to

$$v_e \frac{dC_e}{dt} = K^{trans}\left(C_p - \frac{C_e}{v_e}\right) - \lambda C_e \tag{S.4}$$

and, assuming that the initial concentration of contrast agent in the EES is zero ($C_e(0) = 0$), from Eq. (1), the tissue CA concentration results in

$$C_t(t) = v_p C_p(t) + K^{trans} \int_0^t C_p(\tau) \exp\left(-\left(\lambda + K_{ep}\right)(t-\tau)\right) d\tau\,. \tag{S.5}$$

Using the formalism of the differential algebra approach introduced in Section 2.4, we can analyze the structural identifiability of the mLTK model. We rewrite Eq. (S.4) in the following form:

$$\begin{cases} v_e \dot{x} = K^{trans}\left(u - \dfrac{x}{v_e}\right) - \lambda x \\ y = v_e x + v_p u \end{cases} \tag{S.6}$$

where $y(t)$ is the observable concentration of the contrast agent in the tissue $C_t(t)$ and $u(t)$ is the external input given by the concentration of contrast agent in the plasma compartment (VIF). Differentiating the second equation and combining it with the first one, we can write the differential equation for $y(t)$ in the form

$$\dot{y} + a_1 y + a_2 u + a_3 \dot{u} = 0$$

where the coefficients a_i are given by

$$\begin{cases} a_1 = \dfrac{1}{v_e}\left(\dfrac{K^{trans}}{v_e} + \lambda\right) \\ a_2 = -\left(K^{trans} + \dfrac{v_p}{v_e}\left(\dfrac{K^{trans}}{v_e} + \lambda\right)\right) \\ a_3 = -v_p \end{cases} \tag{S.7}$$

We observe that $a_2, a_3 < 0$, while $a_1 > 0$. Rearranging the terms in Eq. (S.7), we get

$$\begin{cases} K^{trans} = a_3 a_1 - a_2 \\ v_p = -a_3 \\ \lambda = a_2 - a_3 a_1 v_e + a_1 v_e^2 \end{cases} \tag{S.8}$$

From system (S.8) we notice that the parameters K^{trans} and v_p are structurally identifiable, while λ and v_e are structurally non-identifiable. The parameters λ and v_e are connected with the relationship

$$a_2 = \lambda + a_3 a_1 v_e - a_1 v_e^2$$

which has an infinite number or solution for (v_e,λ). This suggests a structurally identifiable reparameterization of mLTK model. Defining a new parameter $\bar{K}^{trans} = \dfrac{K^{trans}}{\lambda}$ and a new time scale $\tau = t\lambda$, system (S.6) reads

$$\begin{cases} v_e \dot{x} = \bar{K}^{trans}\left(u - \dfrac{x}{v_e}\right) - x \\ y = v_e x + v_p u \end{cases} \tag{S.9}$$

where $\dot{x} := \dfrac{dx}{d\tau}$. Repeating the same differential algebra approach introduced above, from system (S.9) we obtain

$$\begin{cases} \bar{K}^{trans} = a_1 a_3 - a_2 \\ v_e = \dfrac{1 + \sqrt{1 + 4a_1(a_1 a_3 - a_2)}}{2a_1} \\ v_p = -a_3\,, \end{cases}$$

i.e., the reparametrized mLTK model is structurally identifiable. Here, for v_e to be well-defined we need $(a_1 a_3 - a_2) \geq 0$, which always holds as $(a_1 a_3 - a_2) = \bar{K}^{trans}$ and the four parameters of the mLTK model have to be positive.

Concerning the practical identifiability of the mLTK model, we use the method described in Section 2.4.2, considering the three cases of study (AA), (RA), and (RR), described in 3.2, and the three different time-enhancement profiles observed in CA evolution. For the purpose of this study and in analogy with LTK analysis, we report the results for the Type I time-enhancement curve and for the parameters K^{trans} and λ. Fig B collects the results concerning practical identifiability of these parameters. Starting from the first three

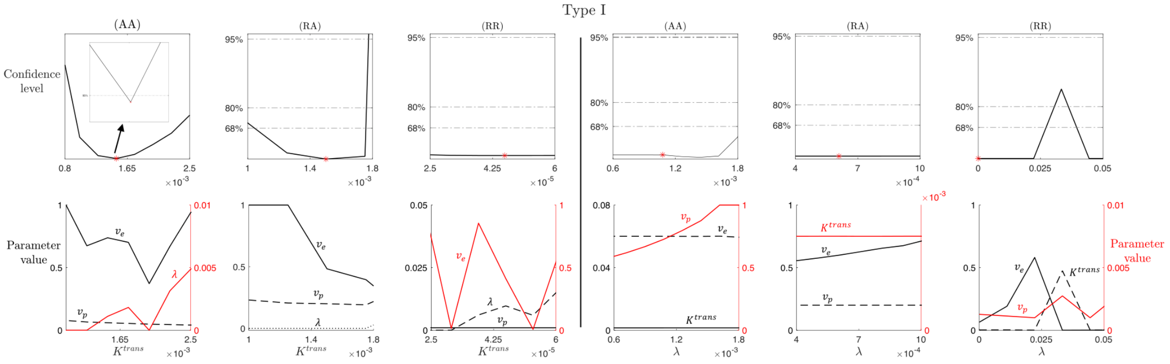

Figure B: **K^{trans} and leakage (λ) practical identifiability for mLTK model and Type I enhancement curve.** Top row: profile likelihood and confidence levels at 68%, 80%, and 95% for the parameter K^{trans} (columns one to three) and λ (columns four to six) in the (AA), (RA), and (RR) case for the Type I enhancement curve. Insets in the first and fourth subplots show a zoom of the region around the best-fitted value $\hat{K}^{trans}$ and $\hat{\lambda}$ (red markers), respectively. Bottom row: compensating profiles of the parameters v_e, v_p, and λ with respect to variation of K^{trans} around its best-fitted value (columns one to three) and of the parameters K^{trans}, v_e, and v_p with respect to variation of λ around its best-fitted value (columns four to six). Variation of $\pm 50\%$ around the optimal values of K^{trans} and λ are considered. Two colors are for two different y-axis: black curves refer to the left y-axis and red curves to the right y-axis. Different line styles are used to distinguish curves referring to the same y-axis. For each curve, the name of the corresponding parameter is indicated above the line in the same color.

columns, referring to the parameter K^{trans} in the (AA), (RA), and (RR) cases, we notice that the results are analogous to the ones shown in Fig 4. In fact, if the artificial data set used in (AA) allows to recover the practical identifiability of K^{trans} (identified by the parabola-like profile with a unique minimum in the optimal value $\hat{K}^{trans}$ and a finite confidence region for the 95% confidence level), (RA) case shows a finite confidence region only for the 68% confidence level, while for (RR) case it is not possible to define the lower and upper bounds of the confidence region. Thus, the parameter K^{trans} is practically non-identifiable, especially when real GBM data and individual-based estimation of the VIF are considered. Looking instead at columns four to six, we notice that none of the cases demonstrate identifiability for λ. In all cases, we observe that it is not

possible to define the lower and upper bounds of the confidence region for all the considered confidence levels. This is in agreement with the results about structural identifiability of mLTK model. In fact, λ is a structurally non-identifiable parameter for mLTK and, from the theory about model identifiability, we know that structural non-identifiability implies practical non-identifiability.
