# Supplementary material for: Structural and practical identifiability of contrast transport models for DCE-MRI
Source: PLoS Comput Biol. 2024 May 15;20(5):e1012106. doi: 10.1371/journal.pcbi.1012106 (PMC11132485; doi:10.1371/journal.pcbi.1012106)
Supplement: S3 Text — It contains Tables A and B and Figs A, B, C, D. (PDF) [file pcbi.1012106.s003.pdf]

S3 Supplementary data for the LTK Model

Here, we collect additional data and results concerning the analysis of the LTK model proposed in these notes. Table A summarizes the best values for LTK parameter obtained from the data fitting based on a PS algorithm for the three types of CA time-enhancement curves in the three cases of study (AA), (RA), and (RR). Table B summarizes the coordinates (X, Y, Z) of the voxels of the DCE image from which the three different enhancement pattern for the CA concentration were obtained for the (RR) study in the GBM and breast cancer tissue. Figs A and B collect the results of the practical identifiability analysis for the parameters K^{trans} and λ for Type II and III CA time-enhancement curves. In both Figs, Type II and Type III time-enhancement profile results are in line with the ones obtained for Type I in Figs 4 and 5. For the artificial dataset and population-based estimation of the VIF ((AA) case), K^{trans} and λ practical identifiability is confirmed with finite confidence region for the 95% confidence level, while it is not possible to define lower/upper (or both) bound of the confidence region for the given confidence levels when an individual-based estimation of the VIF in both (RA) and (RR) cases is considered.

Case	Type	K^{trans} (s^{-1})	v_e	v_p	λ (s^{-1})
(AA)	I	0.0022	0.6193	0.0499	0.0104
(AA)	II	0.009	0.5001	0.1001	0.001
(AA)	III	0.03	0.05	0.1	0.001
(RA)	I	0.0025	0.4973	0.1	0.001
(RA)	II	0.009	0.5	0.1	0.001
(RA)	III	0.8018	0.0326	0.8684	0
(RR)	I	0.000449	0.0106	0.001	10^{-6}
(RR)	II	0.1396	0.2315	0.0033	0.000482
(RR)	III	0.1793	0.001	0.1632	0

Table A: Best parameter values obtained with PS algorithm for the three types of CA time-enhancement curves and in the three cases of study.

Case	Type	X	Y	Z
GBM	I	103	53	10
	II	32	66	7
	III	38	65	8
Breast	I	80	112	5
	II	78	118	5
	III	81	117	5

Table B: X, Y, and Z coordinates of the DCE image voxels for the three different CA concentration patterns in the GBM and breast cancer tissue cases of study.

S3.1 RR - Breast cancer tissue

In this Section, we analyze the practical identifiability of the LTK model in breast cancer tissue under the (RR) case. Fig C shows the LTK model fit to the data using Type I, II, and III time-enhancement curves. For breast cancer tissue, the leakage compartment introduced with the LTK and mLTK models calibrates to zero, and, thus, it is not necessary. This indicates that leakage in breast cancer is less important than in GBM. Application to GBM, where large regions of necrosis with slow contrast uptake were the original motivation for including a leakage compartment [1,2]. As breast tumors are often highly perfused [3–5], it follows that leakage often calibrates to 0. Despite this, we analyzed practical identifiability of K^{trans} and λ, varying the latter in the neighborhood of 0 to observe how the profile likelihood and compensating profiles evolve. Fig D shows the results about practical identifiability for K^{trans} and λ for Type I time-enhancement curve in the (RR) case when breast cancer tissue is considered. As shown in the last columns of Figs 4 and 5, also here both K^{trans} and λ result practically non-identifiable parameters, independently from the characteristics of the considered

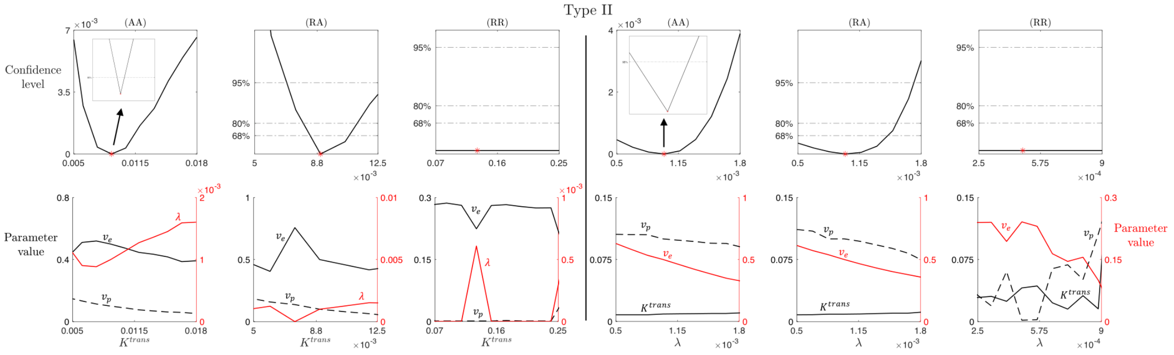

Figure A: K^{trans} **and leakage (**λ**) practical identifiability for LTK model and Type II enhancement curve.** Top row: profile likelihood and confidence levels at 68%, 80%, and 95% for the parameter K^{trans} (columns one to three) and λ (columns four to six) in the (AA), (RA), and (RR) case for the Type II enhancement curve. Insets in the first and fourth subplots show a zoom of the region around the best-fitted value $\hat{K}^{trans}$ and $\hat{\lambda}$ (red markers), respectively. Bottom row: compensating profiles of the parameters v_e, v_p, and λ with respect to variation of K^{trans} around its best-fitted value (columns one to three) and of the parameters K^{trans}, v_e, and v_p with respect to variation of λ around its best-fitted value (columns four to six). Variation of $\pm 50\%$ around the optimal value of K^{trans} and λ are considered. Two colors are for two different y-axis: black curves refer to the left y-axis and red curves to the right y-axis. Different line styles are used to distinguish curves referring to the same y-axis. For each curve, the name of the corresponding parameter is indicated above the line in the same color.

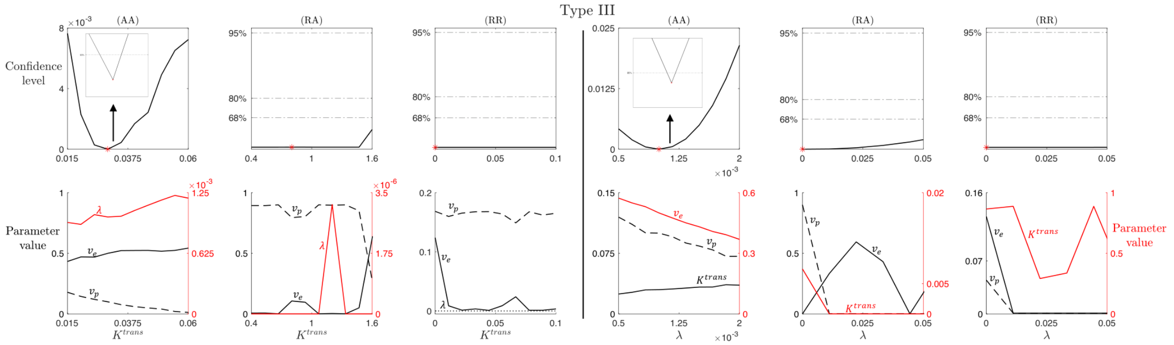

Figure B: K^{trans} **and leakage (**λ**) practical identifiability for LTK model and Type III enhancement curve.** Top row: profile likelihood and confidence levels at 68%, 80%, and 95% for the parameter K^{trans} (columns one to three) and λ (columns four to six) in the (AA), (RA), and (RR) case for the Type III enhancement curve. Insets in the first and fourth subplots show a zoom of the region around the best-fitted value $\hat{K}^{trans}$ and $\hat{\lambda}$ (red markers), respectively. Bottom row: compensating profiles of the parameters v_e, v_p, and λ with respect to variation of K^{trans} around its best-fitted value (columns one to three) and of the parameter K^{trans}, v_e, and v_p with respect to variation of λ around its best-fitted value (columns four to six). Variation of $\pm 50\%$ around the optimal value of K^{trans} and λ are considered. Two colors are for two different y-axis: black curves refer to the left y-axis and red curves to the right y-axis. Different line styles are used to distinguish curves referring to the same y-axis. For each curve, the name of the corresponding parameter is indicated above the line in the same color.

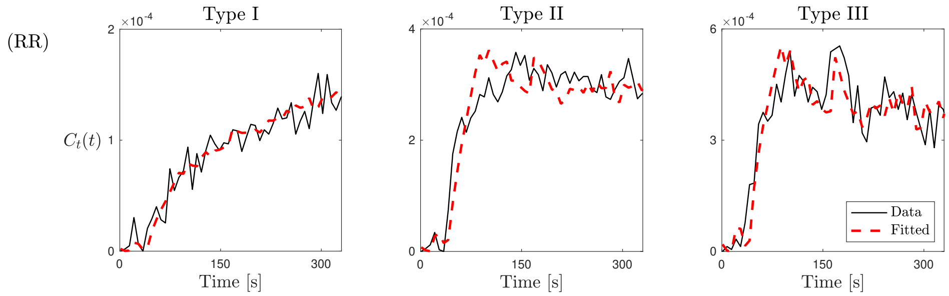

Figure C: **Best fitting of the CA evolution with the LTK model** (9)**.** The three types of CA time-enhancement curves (columns) are shown or the (RR) case for the breast cancer tissue.

Type I

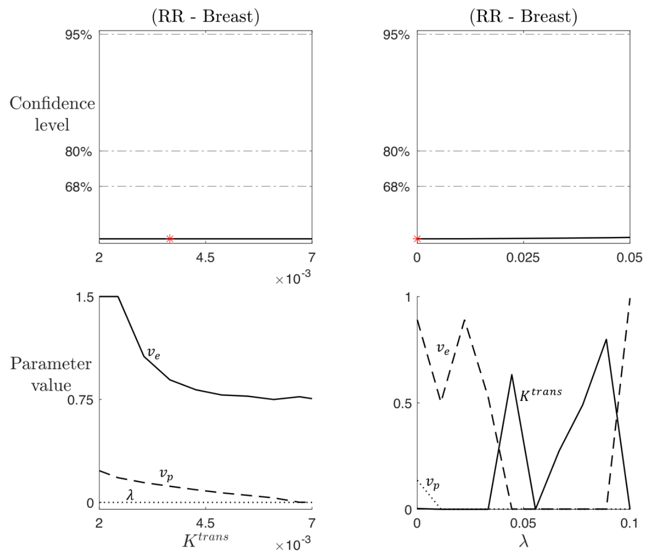

Figure D: K^{trans} **and leakage (λ) practical identifiability for LTK model and Type I enhancement curve for breast cancer tissue.** Top row: profile likelihood and confidence levels at 68%, 80%, and 95% for the parameter K^{trans} (first column) and λ (second column) in the (RR) case for breast cancer tissue and for the Type I time-enhancement curve. Bottom row: compensating profiles of the parameters v_e, v_p, and λ with respect to variation of K^{trans} around its best-fitted value (first column) and of the parameters K^{trans}, v_e, and v_p with respect to variation of λ around its best-fitted value (second column). Red markers indicate the best-fitted values $\hat{K}^{trans}$ and $\hat{\lambda}$. Variation of $\pm 50\%$ around the optimal value of K^{trans} and λ are considered. Different line styles are used to distinguish the curves referring to the three parameters. For each curve, the name of the corresponding parameter is indicated above the line.

tissue. It is not possible, in fact, to define lower and upper bounds for the confidence regions for any of the considered confidence levels.

References

[1] Sahoo, P, Rathore, RK, Awasthi, R, Roy, B, Verma, S, Rathore, D, et al. Subcompartmentalization of extracellular extravascular space (EES) into permeability and leaky space with local arterial input function (AIF) results in improved discrimination between high-and low-grade glioma using dynamic contrast-enhanced (DCE) MRI. J Magn Reson Imaging 2013; 38:677–88.

[2] Jain, K, Sahoo, P, Tyagi, R, Mehta, A, Patir, R, Vaishya, S, et al. Prospective glioma grading using single-dose dynamic contrast-enhanced perfusion MRI. Clin Radiol. 2015; 70:1128–35.

[3] Syed, AK, Whisenant, JG, Barnes, SL, Sorace, AG, and Yankeelov TE. Multiparametric analysis of longitudinal quantitative MRI data to identify distinct tumor habitats in preclinical models of breast cancer. Cancers 2020; 12:1682.

[4] Kazerouni, AS, Hormuth, DA, Davis, T, Bloom, MJ, Mounho, S, Rahman, G, et al. Quantifying tumor heterogeneity via MRI habitats to characterize microenvironmental alterations in HER2+ breast cancer. Cancers 2022; 14:1837.

[5] Barnes, SL, Sorace, AG, Loveless, ME, Whisenant, JG, and Yankeelov, TE. Correlation of tumor characteristics derived from DCE-MRI and DW-MRI with histology in murine models of breast cancer. NMR in Biomedicine 2015; 28:1345–56.
